# Surgical therapy and outcome of descending necrotizing mediastinitis in Chinese: a single-center series

**DOI:** 10.3389/fmed.2023.1337852

**Published:** 2024-01-11

**Authors:** Zhewei Zhao, Dongjie Ma, Yuan Xu, Chao Guo, Shanqing Li, Jian Wang, Mu Wang, Yingzhi Qin, Hongsheng Liu

**Affiliations:** ^1^Department of Thoracic Surgery, Peking Union Medical College Hospital, Chinese Academy of Medical Sciences and Peking Union Medical College, Beijing, China; ^2^Department of ENT, Peking Union Medical College Hospital, Chinese Academy of Medical Sciences and Peking Union Medical College, Beijing, China; ^3^Department of Stomatology, Peking Union Medical College Hospital, Chinese Academy of Medical Sciences and Peking Union Medical College, Beijing, China

**Keywords:** descending necrotizing mediastinitis, video-assisted thoracic surgery, transcervical drainage, transthoracic drainage, surgical treatment

## Abstract

**Background:**

Descending Necrotizing Mediastinitis (DNM) is an acute and often fatal infection that affects the neck and mediastinum. DNM treatment consists of broad-spectrum antibiotics, early diagnosis, and surgical debridement with multidisciplinary cooperation. However, owing to the rarity and complexity of this disease, the mortality rate is high. This retrospective study analyzed a single-center experience of managing DNM in Chinese patients over the last 10 years.

**Methods:**

A single-center, retrospective, observational, and descriptive study was conducted on 31 patients with DNM at Peking Union Medical College Hospital from 2012 to 2022. Case report forms were used to collect data which were then analyzed with a focus on surgical management and outcomes.

**Results:**

This study examined the outcomes of 31 patients diagnosed with DNM at our hospital. The most common comorbidities on admission were hypertension (48%) and diabetes mellitus (42%). The degree of diffusion of DNM according to Endo’s classification was classified as follows: type I in 7 patients (22.6%), type IIA in 5 (16.1%), and type IIB in 19 patients (61.3%). Among these patients, 13 (41.9%) were found to have a single microbial infection, while 16 (51.6%) were found to have polymicrobial infections. In all cases, neck drainage was performed via cervicotomy, with multiple drains (64.5%) and vacuum sealing drainage (VSD) (35.5%). Mediastinal drainage was performed via a cervical mediastinotomy (51.6%), video-assisted thoracic surgery (VATS) (41.9%), or thoracotomy (6.5%). The 30-day mortality rate was 25.8% and 24.0 days of the average length of hospital stay.

**Conclusion:**

Early accurate diagnosis and timely intervention have been shown to be correlated with a positive prognosis. Cervicothoracic CT (computed tomography) is essential for the diagnosis, staging, and evaluation of the optimal surgical treatment. Cervicotomy and video-assisted thoracoscopic surgery with percutaneous drainage is effective, even in advanced cases. Additionally, the application of VSD in cervical incision did not improve prognosis but may shorten the length of ICU (intensive care unit) and hospital stays.

## Background

Descending necrotizing mediastinitis (DNM) a rapidly progressing infection of the mediastinum and neck has a high mortality rate. DNM was first reported by Pearse (1) and most cases develop from oropharyngeal or cervical infections and spread to the chest cavity (2). The affected patients can develop an adverse general condition with sepsis, especially when a mediastinal abscess develops in the mediastinal connective tissues and causes tissue necrosis (2, 3).

In 1983, Estrera et al. proposed the following DNM diagnostic criteria (4): (1) presentation of severe infection symptoms, (2) identification of distinct radiographic features, (3) confirmation of necrotizing mediastinal infection through surgical intervention, and (4) detection of an oropharyngeal/cervical infection with a clear descending necrotizing mediastinitis relationship. Depending on the anatomical scope, Endo et al. (5) established a classification of DNM as follows: type I, where the infection is confined to the upper mediastinum (above the carina); type IIA, where infection extends to the lower anterior mediastinum; and type IIB, where the infection spreads to both the posterior and anterior mediastinum.

Broad-spectrum antibiotics, early diagnosis, and surgical debridement with multidisciplinary cooperation are the cornerstones of treatment. Nevertheless, owing to its rarity and complexity, the mortality rate is high with a range of 11–40% (2–4). However, appropriate guidelines and management protocols for this severe disease are still lacking. In this retrospective study, we analyzed our single-center experience in managing this rare and life-threatening disease over the last 10 years.

## Materials and methods

This report followed the Strengthening the Reporting of Observational Studies in Epidemiology (STROBE) reporting guideline (6). A single-center, retrospective, observational, and descriptive study was conducted on all cases of DNM treated at Peking Union Medical College Hospital from January 2012 and July 2022. The inclusion criteria were: patients who were diagnosed with DNM according to the definition proposed by Estrera et al. (4). Computed tomography (CT) was used to identify the spread of infection. Patients with acute mediastinitis resulting from non-descending causes were excluded from the study. A total of 31 patients were included in this study. Individual patient data were recorded in an electronic format using a case report form (CRF). The items including physical status (age, sex, and comorbidities) and clinical data (etiology of DNM, initial symptoms, radiological examination type at the diagnosis, Endo classification of DNM, identified pathogens, antibiotics, operation data, drainage data, presence of combined tracheostomy, hospitalization period, morbidity, and mortality) were reviewed using the CRF.

The means and standard deviations of continuous variables were reported, while nominal variables were reported as counts and percentages. The statistical significance of the factors was assessed using either Pearson’s chi-square test or Fisher’s exact test. Furthermore, a Cox proportional hazards model was employed to perform multivariate analysis and evaluate the prognostic significance of the identified factors. *P* < 0.05 was the significance threshold. SPSS (version 19.0, SPSS software, Munich, Germany) was used for statistical analysis.

## Results

### Characteristics on admission

Thirty-one patients with DNM were treated at our hospital between January 2012 and July 2022. Seven patients (22.6%) were female and twenty-four (77.4%) were male. Patients’ ages ranged from 28 to 72 years (median, 57 years). Table 1 displays the clinical background characteristics of the patients at admission. Of the 31 patients, hypertension was the most frequent comorbidity (48%), followed by diabetes mellitus (DM) (42%) and coronary heart disease (16%).

**TABLE 1:** Characteristics of the patient group.

Variables	Number	Percentage
**Gender**		
Male	24	77.4
Female	7	22.6
Median of age (years)	57.19 (28–72)	
**Comorbidities**		
Diabetes mellitus	13	41.9
Hypertension	15	48.4
Coronary heart disease	5	16.1
Cerebrovascular disease	3	9.7
Autoimmune disease	2	6.5
Steroid use	1	3.2
Others	7	22.6
**Symptoms**		
Pain	30	96.8
Dyspnea	19	61.3
Fever	18	58.1
Swelling	17	54.8
Dysphagia	5	16.1
Impaired consciousness	2	6.5
Others	6	19.4

The frequently observed symptoms on admission were pain (96.8%), maxillofacial and neck pain (87.1%), chest pain (12.9%), dyspnea (61.3%), fever (58.1%), neck swelling (54.8%), dysphagia (16.1%), and impaired consciousness (6.5%).

### Etiology

The most common source of DNM were peritonsillar/retropharyngeal infections (38.7%) and odontogenic infections (45.2%) (Table 2). Some rare causes included trauma and thyroid abscess. On average, a second diagnosis of DNM occurred 5.6 days after the initial diagnosis of the infection’s source (range, 1–13 days).

**TABLE 2:** Etiology of DNM.

Etiology of DNM	Number	Percentage
Peritonsillar/retropharyngeal abscess	11	35.48
Odontogenic	14	45.16
Parotitis	1	3.23
Traumatic	2	6.45
Thyroid abscess	1	3.23
Others	2	6.45

### Diagnostics

An otolaryngological examination was performed on all patients, and they subsequently underwent cervicothoracic CT to confirm the clinical diagnosis and evaluate the extent of the infection. Among them, 8 underwent contrast-enhanced CT and 23 underwent plain CT (Table 3). Imaging features included pleural effusion (77.4%), pneumomediastinum (64.5%), pericardial effusion (51.6%), enlarged mediastinal lymph nodes (38.7%), low-density mediastinal images (35.5%), increased density of mediastinal fat (19.4%), and pericardial thickening (6.5%).

**TABLE 3:** Computed tomography (CT) scan of the patient group.

Variables	Number	Percentage
**CT scan**		
Enhanced CT	8	25.81
Plain CT	23	74.19
**Imaging features**		
Pleural effusion	24	77.42
Pneumomediastinum	20	64.52
Pericardial effusion	16	51.61
Enlarged mediastinal lymph nodes	12	38.71
Low density images in the mediastinum	11	35.48
Increase in the density of mediastinal fat	6	19.35
Pericardial thickening	2	6.45

The degree of diffusion of DNM according to Endo’s classification was as follows: type I, 7 patients (22.6%), and type II, 24 (77.4%). Out of the 24 type II cases, 5 (16.1%) were classified as extending to the lower anterior mediastinum (type IIA), while 19 (61.3%) were classified as extending to both the posterior and anterior mediastinum (type IIB).

### Microbiology

Microbiological examinations were conducted, and bacterial and fungal infections were detected in 29 patients (93.5%) as shown in Table 4. Of these, 13 (41.9%) revealed a single microbial infection, while 16 (51.6%) showed polymicrobial infections. In two cases, more than three pathogens were identified by microbiological examination. In 2 patients (6.5%), anaerobic and aerobic cultures exhibited no growth, which could be attributed to either unsuccessful isolation of bacteria or prior antibiotic treatment.

**TABLE 4:** Microbiology of DNM.

Variables	Number	Percentage
**Number of identified pathogens in one case**		
None	2	6.45
One	13	41.94
Two	12	38.71
Three	2	6.45
More than three	2	6.45
**Identified pathogens**		
Gram-positive Cocci	23	
Gram-negative Cocci	1	
Gram-positive Bacilli	3	
Gram-negative Bacilli	17	
Yeasts	7	

Among these 29 cases, *Streptococcus* spp. were the most commonly identified bacteria (55.2%), followed by *Acinetobacter baumannii* (20.7%), *Klebsiella pneumoniae* (17.2%), 2 Gram-negative bacilli. Fungal infections were identified in 6 patients (20.7%) (*Candida tropicalis* and *Candida albicans*).

### Treatment

Once a clinical diagnosis of mediastinitis is suspected in the emergency department, broad-spectrum antibiotic therapy should be initiated empirically after blood culture. In this study, patients were initially administered antibiotics, including a third-generation cephalosporin, amoxicillin/clavulanic acid, carbapenems, or vancomycin combined with metronidazole. The treatment was later adjusted according to the results of the microbiological examination of the harvested samples or blood culture.

Surgery to drain and collect mediastinal and neck secretions was performed on all patients. The median time to surgical drainage following DNM diagnosis was 0 day (0–7, 1.0 days in average). Among these patients, 18 patients (58.1%) had undergone surgery on the day of the DNM diagnosis. Ten patients (32.3%) underwent surgery 1–3 days after the diagnosis, whereas the remaining three (9.7%) underwent surgery four or more days later.

Of these 31 patients, eight (25.8%) were treated with simple cervical incision and drainage in local hospitals before being transferred to us. All eight patients underwent surgery in our hospital because of progressive mediastinal infection. In all cases, drainage of the neck was carried out via cervicotomy. Multiple drains (64.5%) and vacuum-sealing drainage (VSD) (35.5%) were applied to cervical incisions. Mediastinal drainage was performed via a cervical mediastinotomy (51.6%), video-assisted thoracic surgery (VATS) (41.9%), or thoracotomy (6.5%). in all cases. Ten patients (32.3%) underwent reoperation, and the average number of operations was 1.5 times (1–4 times). Percutaneous drainage of the mediastinum or thorax in 16 patients (51.6%) was performed via ultrasound or CT-guided for recurrent/remnant abscesses. Tracheostomy was performed in 21 (67.7%) patients.

### Outcome

The average length of hospital stay was 24.0 days (range: 1–68 days) with 10.5 days (range: 0–25 days) of the average length of intensive care unit stay. The 30-day mortality rate was 25.8% (*N* = 8). Five of these eight patients died as a result of septic shock and organ failure. One patient died of disseminated intravascular coagulation and intracerebral hemorrhage. The other two patients who presented with DNM Endo Type IIB abandoned treatment because of financial constraints.

## Discussion

Descending necrotizing mediastinitis is a life-threatening condition characterized by the inflammation of the mediastinum extending from the neck. Some researches revealed that the incidence of DNM has increased in recent years (7). However, the incidence rate of DNM with deep neck infections has been reported to be between 1.5 and 3.5% (8). Surgical therapy is the mainstay of treatment for DNM; however, there are currently no high-evidence guidelines or published articles on the treatment of DNM. In this single-center series, we analyzed the data of 31 patients who underwent surgery in the last 10 years to determine the outcomes of this treatment.

In our study, we found that the incidence of DNM was higher in males (77.4%) than in females (22.6%), which is consistent with the findings of other authors (9, 10). However, some authors reported that there were no differences between the genders (11). The mean age was approximately 57 years (range, 28–72 years), which is comparable to data from another study conducted in an Asian population (10) and slightly higher than the 50 years reported in other literature (12, 13). Hypertension (48%) and DM (42%) were the most commonly observed comorbidities. Since hypertension and DM are major comorbidities in China and most of our patients had a low socioeconomic status with poor awareness of medical care, the proportion of hypertension and DM among these patients was greater than that in some previous studies (hypertension 18–33%, DM 18–28%) (10, 12).

Descending necrotizing mediastinitis is most often caused by bacterial spread from a nearby infection, such as Ludwig’s angina, which is an infection of the submandibular space, usually secondary to infection of the second or third lower molar (14). We found the highest frequency of odontogenic infections (45.2%), followed by peritoneal and retropharyngeal infections (38.7%). Rare causes including parotitis (3.2%) and thyroid abscess (3.2%). These findings are consistent with those of some studies showing odontogenic infections as the main cause (15). However, recent studies have reported a decreasing tendency from odontogenic causes to more frequent pharyngeal causes (2, 7, 16). This change may be due to improvements in dental care technology and greater focus on oral hygiene and odontogenic diseases among patients. Post-traumatic infection was observed in two cases. One patient had a traumatic infection of the cervical internal fixations, further resulting in DNM, and another developed a cervical hematoma due to trauma, which finally led to suppuration and necrosis. Other less common causes of DNM include cervical lymphadenitis, traumatic endotracheal intubation, and jugular intravenous drug use/abuse (17).

Early accurate diagnosis and timely intervention have been shown to be correlated with a positive prognosis. Pain (96.8%) was found to be the common clinical presentation, followed by dyspnea (61.3%), fever (58.1%), swelling (54.8%), and dysphagia (16.1%). Patients usually present with mild non-specific symptoms at an early stage. In our study, most patients self-medicated with antibiotics at home or visited a nearby clinic, leading to a late diagnosis. If adequate treatment measures are not initiated at an early stage, the infection has the potential to rapidly spread into the mediastinum, leading to severe infection, sepsis, and eventually death. In addition to oral examinations and laryngoscopy, cervicothoracic CT is essential for patients with DNM. It can be used for assessing the extent of infection, establishing a diagnosis, and aiding in surgical treatment (18). Regarding radiological signs, pleural effusion (77.4%) was the most common, followed by pneumomediastinum (64.5%), pericardial effusion (51.6%), enlarged mediastinal lymph nodes (38.7%), low-density images in the mediastinum (35.5%), increased mediastinal fat density (19.4%), and pericardial thickening (6.5%), which provided the earliest means of detecting DNM.

Endo classification of DNM can also be evaluated using CT, as mentioned previously. Most of our patients had type II (77.4%), and a few had type I (22.6%). Specifically, type IIA was found in 16.1% of the patients, whereas type IIB was found in 61.3%. Due to delayed medical presentation, the number of patients with type IIB was much higher than that of the other groups in our study. Type IIB disease, which represents a further advanced stage of DNM, showed a higher mortality rate than type I and IIA disease in our study (type I 14.3%, type IIA 20.0%, type IIB 31.6%). Sugio et al. named a new type of IIC DNM, in which the lower mediastinal infection is limited to the posterior mediastinum (10). They reported that type IIC was strongly associated with retrovisceral space infections in the neck and mainly underwent VATS. However, there were no significant differences in the mortality and overall survival of each subtype of type II DNM. Further studies are required to validate this new classification.

Consistent with previously reported studies (19), patients with DNM had mixed anaerobic and aerobic polymicrobial infections. The most common aerobic bacteria include *Streptococcus* species, *Staphylococcus aureus*, and *Klebsiella pneumoniae* (9). The most common anaerobic bacteria were *Peptostreptococcus*, *Bacteroides fragilis*, *Prevotella*, and *Porphyromonas* (20). Therefore, it is necessary to use broad-spectrum antibiotics that can target gram-positive, gram-negative, and anaerobic bacteria before cultures are obtained.

Antibiotic therapy alone is undoubtedly insufficient for the treatment of DNM, and multidisciplinary collaborative surgical treatment from stomatology, otolaryngology, and thoracic surgery departments is necessary. Debridement to evacuate all necrotic tissue and sufficient drainage of the mediastinum are essential parts of surgery. For type I DNM limited to the upper part of the mediastinum, drainage via cervicotomy or video mediastinum copies may be sufficient (12, 21, 22). However, in cases of type II DNM, access via thoracotomy combined with transcervical debridement is often recommended (12, 13, 23). Some researchers prefer median sternotomy and clamshell thoracotomy, which exposes and debrides the entire mediastinum (3, 24, 25). However, osteomyelitis or dehiscence of the sternum and approaches for repeat revision surgeries remain major problems (12). In extensive and infected chest wounds, transplanting vascularized tissues can stabilize the chest wall, finally treating infections and promoting healing (26). Albacete Neto et al. found that using a unilateral pectoralis major muscle flap is safe for managing deep sternal wound infections and sternotomy dehiscences, even for larger defects (14). An increasing number of studies have reported treatment of DNM using video-assisted thoracoscopic surgery. VATS induces less trauma, lower morbidity rates, and a rapid recovery, especially in patients requiring bilateral surgery (22, 27–29). To some extent, VATS can provide a clear view of the surgical field to reduce iatrogenic injuries. Therefore, in this study, VATS was performed in most patients, and only two patients underwent thoracotomy. Some studies have suggested that VATS may not allow sufficient debridement in advanced cases (12, 19, 30). Ultrasound- or CT-guided percutaneous drainage is very effective in managing these small residual inflammatory collections. Some partitioned encapsulated fluid collections may require two or more percutaneous drainages to obtain satisfactory results. Undoubtedly, each technique presents potential advantages and disadvantages. Choosing personalized surgical treatments based on the patient’s condition, disease severity, and the surgeon’s expertise is important (26). This careful selection aims to minimize complications, reduce the need for reoperations, and lower mortality rates.

Vacuum sealing drainage is widely used in clinical applications, including osteomyelitis and the management of infected and burn wounds (31). With the help of negative pressure suction and normal saline irrigation, VSD can provide sufficient drainage of inflammatory substances and promote granulation tissue. Several studies have reported that vacuum-assisted drainage is a simple, safe, and effective method for treating DNM (25, 32). Eleven patients underwent VSD during a cervical incision after radical debridement. Interestingly, although there was no difference in 30-day mortality between two groups, application of VSD shortened both the length of ICU stay (5.9 days vs. 13.1 days, *P* = 0.14) and the length of hospital stay (15.6 vs. 28.1 days, *P* = 0.018). This may be due to the formation of a closed negative-pressure microenvironment that improves the drainage effect. Further studies involving more patients are required to investigate the safety and efficacy of VSD in patients with DNM.

Several limitations warrant consideration in interpreting our findings. Primarily, the retrospective nature of our study poses inherent constraints, limiting the depth of data collection and the potential for biases despite rigorous efforts to minimize them. The relatively small sample size of 31 patients from a single center restricts the generalizability of our conclusions to a broader population. Furthermore, given the complexity of DNM, we acknowledge the presence of inherent biases that remain unaccounted for in our analysis. Variables beyond our control, variations in treatment protocols, and unmeasured confounders could have influenced outcomes, necessitating a cautious interpretation of our results. Although DNM is rare, we still hope for larger-scale studies in the future to validate and further elucidate our observational results.

## Conclusion

Descending necrotizing mediastinitis is a rare life-threatening disease. This retrospective study revealed the surgical therapy and outcomes in a single-center series with a 30-day mortality rate of 25.8%. A favorable outcome is often associated with immediate detection, diagnosis, and treatment. Cervicothoracic CT is essential for the early diagnosis, clinical staging, and evaluation of optimal surgical therapeutic options. In our study, cervicotomy and video-assisted thoracoscopic surgery combined with percutaneous drainage were effective even in advanced cases. Although it does not improve prognosis, the application of VSD in cervical incisions can shorten both the length of ICU stay and hospital stay.

## Data availability statement

The raw data supporting the conclusions of this article will be made available by the authors, without undue reservation.

## Ethics statement

The studies involving humans were approved by the Peking Union Medical College Hospital Ethics Review Committee (No. I-22PJ174). The studies were conducted in accordance with the local legislation and institutional requirements. The Ethics Committee/Institutional Review Board waived the requirement of written informed consent for participation from the participants or the participants’ legal guardians/next of kin because this study is a retrospective analysis that utilizes existing medical records data, without involving any changes in medical interventions or the collection of biological specimens. Patient information will be de-identified, and an exemption from informed consent will be sought.

## Author contributions

ZZ: Conceptualization, Data curation, Funding acquisition, Methodology, Writing – original draft. DM: Data curation, Resources, Writing – review and editing. YX: Data curation, Writing – original draft. CG: Data curation, Writing – original draft. SL: Conceptualization, Resources, Supervision, Writing – review and editing. JW: Data curation, Resources, Writing – review and editing. MW: Data curation, Resources, Writing – review and editing. YQ: Conceptualization, Investigation, Resources, Supervision, Writing – review and editing. HL: Conceptualization, Funding acquisition, Investigation, Resources, Supervision, Writing – review and editing.
